# Aggregation of AcMNPV LEF-10 and Its Impact on Viral Late Gene Expression

**DOI:** 10.1371/journal.pone.0154835

**Published:** 2016-05-06

**Authors:** Xiaodong Xu, Xinyu Zhou, Hao Nan, Yu Zhao, Yu Bai, Yanmei Ou, Hongying Chen

**Affiliations:** College of Life Sciences, Northwest A & F University, Yangling, Shaanxi, 712100, P. R. China; Georgia Regents University, UNITED STATES

## Abstract

The Autographa californica multiple nucleopolyhedrovirus (AcMNPV) late expression factor gene *lef-10* has been identified to be required for viral late gene expression by transient expression assay. Our previous work has shown that the gene product LEF-10 can form very stable high-molecular-weight complexes, but the structure and function of the protein remain unknown. In this study, we demonstrated that LEF-10 was essential for the replication of AcMNPV, and its truncated fragment containing amino acid residues 1 to 48 were sufficient to support the virus survival. Our data also suggested that the LEF-10 could spontaneously aggregate to form punctate spots in virus infected *Sf*9 cells at low frequency, and the aggregation of the protein could be induced by LEF-10 over-expression. When the protein aggregated to form punctate spots, soluble LEF-10 proteins were depleted and this could result in the down-regulation of viral late gene expression.

## Introduction

Baculoviruses are insect-specific viruses, mainly infecting Lepidopteran insects. Their gene expression can be divided into three phases: early, late and very late periods. Baculoviruses utilize host RNA polymerase II as well as virus-encoded RNA polymerase to transcribe viral genes during infection [1]. Host RNA polymerase II recognizes baculovirus early promoters, and the virus-encoded RNA polymerase recognizes the late and very late promoters [2,3]. The expression of viral late/very late genes requires the accumulation of viral early gene products including the virus-encoded RNA polymerase.

Autographa californica multiple nucleopolyhedrovirus (AcMNPV), a well-studied baculovirus, encodes about 155 genes [3,4]. Nineteen of these genes have been identified by transient expression assays as late expression factor genes (*lef*) [5,6], which are associated with viral DNA replication and/or late gene transcription. Previous studies have shown that *ie-1* [7], *lef-1* [8], *lef-2* [9], *lef-3* [10], *lef-11* [11], *p143* [12], and *DNApol* [12,13] are essential for viral DNA replication and late gene transcription, and *ie-2* [14], *lef-7* [15] and *p35* [16] are not essential but stimulate DNA replication. The other nine *lef* genes (*lef-4*, *lef-5*, *lef-6*, *lef-8*, *lef-9*, *lef-10*, *lef-12*, *p47* and *pp31*) are thought to be directly involved in baculovirus late gene transcription [3,6], with four of them (*lef-4*, *lef-8*, *lef-9* and *p47*) encoding subunits of the viral RNA polymerase complex [17]. Reports have demonstrated that *lef-6*, *lef-12* and *pp31* are not essential for viral replication [18–20]. Lack of *lef-5* does not affect early gene expression and DNA replication but it can disrupt the expression of a late reporter and the production of progeny viruses [21].

*Lef-10* is thought to be one of the genes positively selected during baculovirus evolution [22], and its homologs are found in the genomes of all Group I and most Group II nucleopolyhedroviruses (NPV) and granuloviruses (GV) [3]. It has been reported that *lef-10* of Bombyx mori nucleopolyhedrovirus (BmNPV) is essential for the viral DNA replication and gene expression [23,24].

Previously, we reported that LEF-10 expressed in *Escherichia coli* and *Spodoptera frugiperda* (*Sf*9) cells formed high-molecular-weight complexes [25]. The complexes were very resistant to the treatment of 6M Urea, or 2% SDS with 100 mM DTT (dithiothreitol). This phenomenon has never been reported for any other LEFs or other baculovirus proteins to the best of our knowledge.

To understand the function and characteristics of LEF-10, we constructed a *lef-10* knockout AcMNPV bacmid, identified the regions involved in the function and aggregation of the protein, and investigated the correlation of LEF-10 aggregation with its function in late gene expression regulation.

## Materials and Methods

### Cells and viruses

*Sf*9 cells were maintained in SFX insect medium (Thermo Scientific HyClone) supplemented with 1% fetal bovine serum (FBS) at 27°C. Recombinant baculoviruses were produced by homologous recombination between co-transfected pTriEx-1.1 or pBAC-5 (Novagen) based plasmid and linearized bacmid [26] using FuGENE HD Transfection Reagent (Promega). Baculoviruses generated by homologous recombination were harvested at 96 hours post transfection. Multiplicities of infection (MOI) used in baculovirus infection were 0.05 for virus amplification and 3 for protein expression.

### Plasmid construction

To repair the *lef-10* function in the *lef-10* knockout bacmid, *egfp* was firstly inserted into pTriEx1.1 between *Eco*RI and *Xho*I sites to make pTriEx/GFP. Then the *lef-10* promoter and coding region (-454 to 235 or -454 to 196) was cloned into pTriEx/GFP between the *Xba*I and *Bam*HI sites and the construct was named as pTriEx/lef10*p*:lef10-GFP and pTriEx/lef10*p*:lef10_1-65_-GFP, respectively.

To increase the expression level of LEF10-GFP and determine the functional region, *lef-10* and the gene fragments for truncated LEF-10 (*lef-10*_*1–52*_, *lef-10*_*1–65*_, *lef-10*_*14–78*_, *lef-10*_*21–78*_ and *lef-10*_*27–78*_) were inserted between the *Nco*I and *Bam*HI sites of pTriEx/GFP separately, and the resultant constructs were named as pTriEx/lef10-GFP, pTriEx/lef10_1-52_-GFP, and so on.

To perform immunofluorescence assay, the *lef-10* promoter and coding region (-147 to 235) was cloned into pBAC-5 between the *Bgl*II and *Xho*I sites. The constructed plasmid was named as pBAC5/lef10*p*:lef10-His, which could produce His-tagged LEF-10 controlled by its intrinsic promoter.

To study the correlation between LEF-10 and late gene expression, the gene of mCherry was first inserted into pTriEx1.1 between the *Nco*I and *Xho*I sites to make pTriEx/mCherry, and then the DNA fragment containing the *p10* promoter and mCherry gene was amplified by PCR and cloned into pTriEx/lef10*p*:lef10-GFP or pTriEx/lef10*p*:lef10_1-65_-GFP between *Xba*I and *Sac*II sites. The constructs generated were named as pTriEx/lef10*p*:lef10-GFP/p10*p*:mCherry and pTriEx/lef10*p*:lef10_1-65_-GFP/p10*p*:mCherry.

### Construction of AcMNPV lef-10 knockout bacmid and generation of LEF-10 repaired viruses

**Fig 1:**
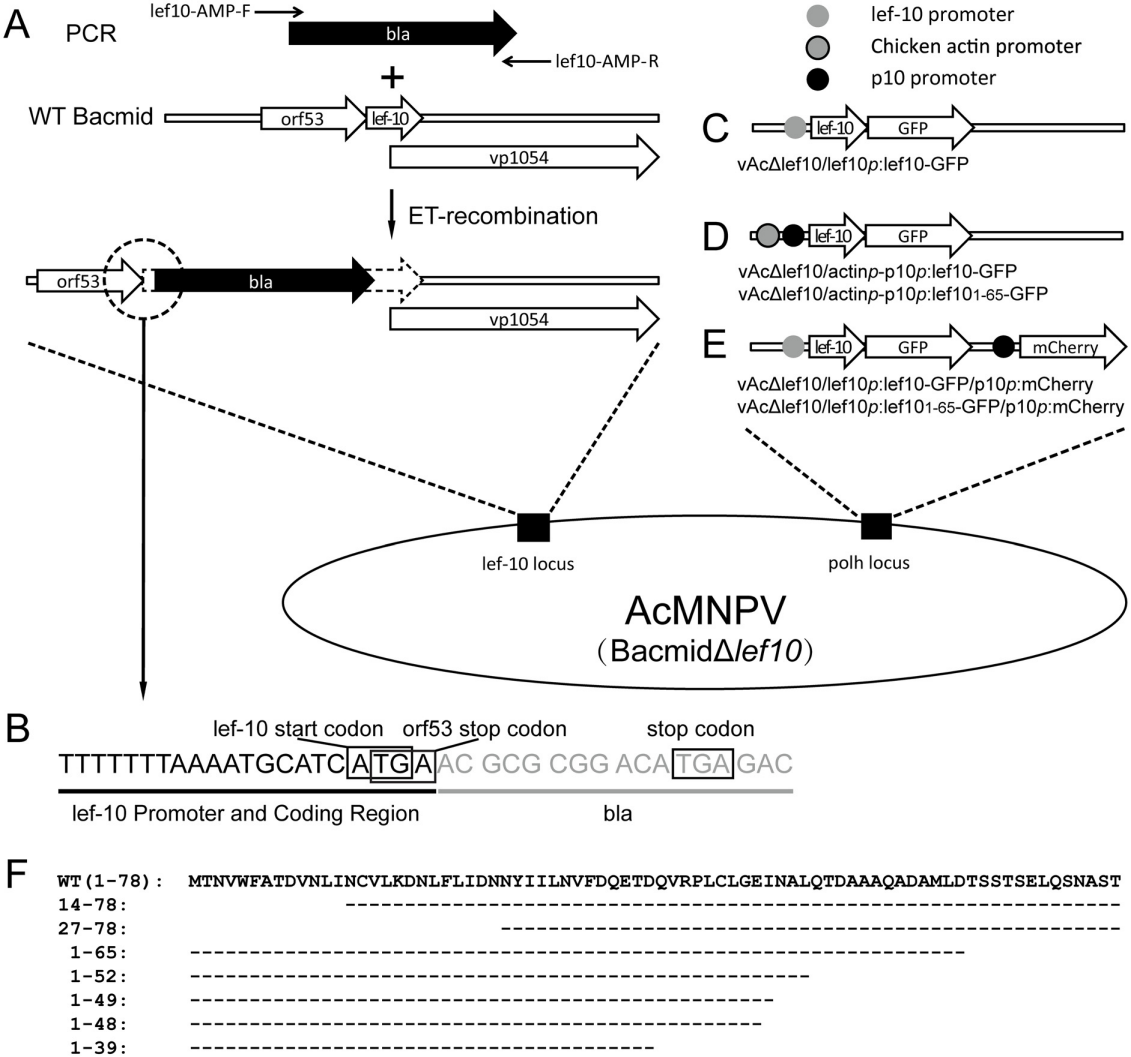
Strategy for construction of BacmidΔ*lef10* and generation of *lef-10* repaired baculoviruses by homologous recombination. (A) Procedure for knockout of *lef-10*. (B) The detailed sequences nearby the start codon of LEF-10 after *bla* insertion. (C) Using LEF10-GFP gene driven by *lef-10* promoter to repair BacmidΔ*lef10*. (D) Using LEF10-GFP gene driven by chicken actin promoter to repair BacmidΔ*lef10*. A tandem strong *p10* promoter was used to over-express LEF-10 in late infection. (E) Using mCherry driven by *p10* promoter as a reporter to reflect virus late gene expression level in *lef-10* repaired baculoviruses. (F) Diagram of LEF-10 truncations used in this study.

To determine whether *lef-10* is essential for virus replication, *lef-10* knockout bacmid was generated in *E*. *coli* strain HS996 using ET-recombination system and bacmid BAC10:KO_1629_ [26,27]. A 11 bp fragment (nucleotide position +5 to +15) within the *lef-10* gene was replaced by ampicillin resistance gene with its stop codon according to the method described by Yu *et al* [23]. Briefly, a pair of primers, lef10-AMP-F (5’-GCCCTGGACATTGAACTCGATTTTAGGCATTTTTTTAAAATGCAATCATGAACGCGCGGACATGAGAC-3’) and lef10-AMP-R (5’-AAATAAATTATCTTTCAGTACACAATTGATCAGGTTGACGTCCGTCGCGATTACCAATGCTTAATCAG-3’), which comprised 50 bp of *lef-10* homologous arm (underlined) and 18 bp of ampicillin resistance gene (beta-lactamases, *bla*), were used to amplify the linear fragment targeting *lef-10* gene by PCR. The amplified linear fragment was transformed into HS996 competent cells containing the Red^®^/ET^®^ plasmid pSC101-BAD-gbaA and bacmid BAC10:KO_1629_ [26,27]. *Lef-10* knockout bacmid (BacmidΔ*lef10*) was generated according to the manufacturer’s instructions (Gene Bridges GmbH, Germany). Strategies for the knockout of *lef-10*, and the generation of LEF-10 repaired baculoviruses, are illustrated in Fig 1A.

To rescue *lef-10* repaired viruses, BacmidΔ*lef10* was co-transfected with pTriEx/lef10*p*:lef10-GFP (Fig 1C), pTriEx/lef10-GFP (Fig 1D) or pTriEx/GFP (as a negative control) into *Sf*9 cells, and recombinant viruses were generated by homologous recombination.

### Growth kinetic analysis of LEF-10 repaired viruses

To compare the one step growth curve of the wild type and LEF-10 repaired recombinant viruses, *Sf*9 cells were infected with the indicated virus at an MOI of 1. Cell culture media were harvested at various time points and analyzed for the presence of infectious virions by endpoint dilution. The averages of the infectious titers from three independent infections were calculated and plotted into graphs.

### Fluorescence microscopy

*Sf*9 cells were infected with recombinant baculoviruses. The expression of fluorescent proteins was observed using fluorescence microscope (Leica DM5000B upright microscope or Olympus CKX41 inverted microscope).

### Immunofluorescence assay

To investigate whether LEF-10 aggregation *in vivo* is caused by GFP fusion, a smaller tag, 8×His instead of GFP, was introduced into the c-terminus of LEF-10, and the protein expression was detected by immunofluorescence assay. Briefly, recombinant baculovirus vAc/lef10*p*:lef10-His was generated by homologous recombination using pBac5/lef10*p*:lef10-His and BacmidΔ*lef10*. *Sf*9 cells (8×10^5^ cells/dish) in 35 mm plate were infected with vAc/lef10*p*:lef10-His at an MOI of 2. After incubation for 24 hours at 27°C, cells were washed 3 times with phosphate-buffered saline (PBS: 50 mM Tris, 150 mM NaCl, pH 7.6). The cells were fixed by adding ice-cold 95% ethanol and incubating for 3 min. Cells were then permeabilized with 0.1% Triton X-100 in PBS at room temperature for 15 min. After washing 3 times with PBS, the cells were incubated overnight at 4°C with anti-His mAb at 1:1000 dilution in PBS. After washing, bound antibody was detected with a goat anti-mouse IgG conjugated to FITC (Beijing CoWin Biotech).

To visualize the cell nuclei, cells were then stained with 10 μg/ml of DAPI in the dark for 30 min. Stained cells were observed under fluorescence microscope.

### Bimolecular fluorescence complementation (BiFC) assay

To study the intermolecular interaction of LEF-10, BiFC assay was performed. pBiFC-VN173 and pBiFC-VC155 were obtained from Addgene (Cambridge, MA). The full-length *lef-10* and *lef-10*_1–65_ was respectively cloned into pBiFC-VN173 between *Eco*RI and *Xba*I sites, and pBiFC-VC155 between *Eco*RI and *Xho*I sites. The constructs were accordingly named as pBiFC/LEF10-VN, pBiFC/LEF10_1-65_-VN, pBiFC/LEF10-VC and pBiFC/LEF10_1-65_-VC. HEK 293T cells monolayer grown in 6-well plates were transfected with 1 μg of each plasmid using calcium phosphate [28], the supernatant was replaced with fresh media at 8 hours post-transfection. The cells were observed at 24 hours post-transfection under fluorescence microscope.

### Flow cytometry

*Sf*9 cells were harvested at 72 hours post infection (hpi) by centrifugation at 300 *g* for 2 min, and then re-suspended in PBS. Data collection was performed on a CyFlow Cube 6 flow cytometer (Partec) at an excitation wavelength of 488 nm. Cells expressing GFP fusion protein were detected in the FL1 channel at emission wavelength of 530 nm. Cells expressing mCherry were detected in the FL2 channel at emission wavelength of 585 nm. Data were collected from at least 2×10^4^ cells for each sample and data analysis was performed offline using FCS Express 4.

### Semi-quantitative RT-PCR

To investigate the effect of LEF-10 aggregation on late gene transcription, semi-quantitative RT-PCR was performed. Total RNAs were isolated with the TRIzol RNA extraction kit (Beijing CoWin Biotech) from 1×10^6^ *Sf*9 cells infected by vAcΔlef10/actin*p-*p10*p*:lef10_1-65_-GFP or vAcΔlef10/actin*p-*p10*p*:lef10-GFP (Fig 1D) at 48 hpi. The RNA samples were then treated with RNase-Free DNaseI (Thermo). Reverse transcription was performed using the 5× All-In-One RT MasterMix (ABM), employing 2 μg total RNA as the template per reaction. The target DNA fragments were then amplified by PCR using the according primer pairs (listed in Table 1). After 25–29 cycles of amplification, the PCR samples were analyzed by 1% agarose gel electrophoresis.

**Table 1:** Primers used in semi-quantitative RT-PCR.

**Gene**	**Primer Name**	**Primer Sequence**
**actin**	ActinF	5’-CAATCTGTCACCTTGGCA-3’
	ActinR	5’-GACAATACAAACTAAGATTTA-3’
**p78/83**	p78/83F	5’-CCTCCACCACCACCACCACCA-3’
	p78/83R	5’-GCGATTTTTTTCGTTTCTAATAGCTTCC-3’
**vp39**	vp39F	5’-CAACGAAAACGCAGTTAACACTATATGC-3’
	vp39R	5’-CGTGTTCGGGTTTGTGGTGTCC-3’
**sod**	sodF	5’-TGAAAGCCATCTGCATCATTAGCG-3’
	sodR	5’-TTGCTCGTGTCGCCATATTCGTG-3’
**odv-e56**	odv-e56F	5’-GATCTTCCTGCGGGCCAAACACT-3’
	odv-e56R	5’-AACAAGACCGCGCCTATCAACAAA-3’
**vp80**	vp80F	5’-CGAACATTACACCGATCAGGACAAAG-3’
	vp80R	5‘-TGATCAATAGTTGTTATGTGCAACCGA-3’
**p10*p*-lef10**	p10*p*-lef10F	5’-GCAATCATGACGAACGTATGGTTC-3’
	p10*p*-lef10R	5’-GGGCGGATCCTTCACCGAGGCACAG-3’

## Results

### AcMNPV lef-10 is essential for virus replication

**Fig 2:**
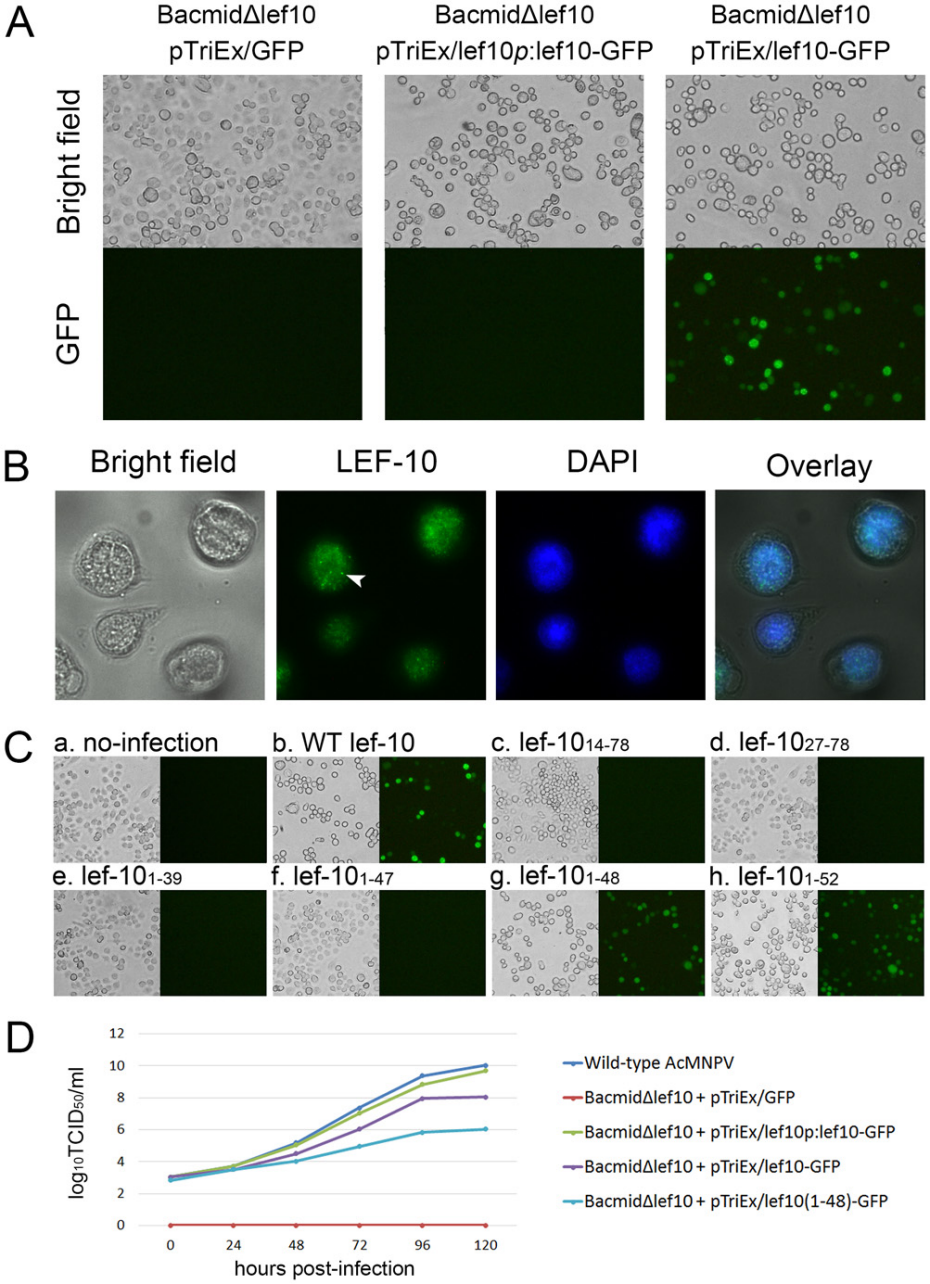
(A) Infection analysis of *lef-10* knockout and repaired AcMNPV. The *Sf*9 cells were infected with cell culture supernatant obtained from cells co-transfected with BacmidΔ*lef10* and the indicated plasmid, and observed at 72 hpi. The negative control using pTriEx/GFP failed to generate infectious viruses, but pTriEx/lef10*p*:lef10-GFP and pTriEx/lef10-GFP repaired BacmidΔ*lef10*. Cells were observed using Olympus CKX41 inverted microscope. (B) Expression and nuclear localization of LEF-10. The cells were infected with the vAc/lef10p:lef10-His recombinant virus and harvested at 24 hpi. After incubation with anti-His primary antibody and a secondary antibody and stained by DAPI, cells were observed using Leica DM5000B upright microscope. The arrowhead indicated a punctate dot formed by LEF-10. (C) Microscopic analysis of *Sf*9 cells infected with supernatant of cell culture co-transfected with BacmidΔ*lef10* and plasmid expressing the indicated gene fragment. Cells were observed under fluorescence microscope using bright light (left side) and blue light (right side) at 72 hpi. (D) Growth curve of AcMNPV with wild-type *lef-10* and *lef-10* repaired AcMNPV.

It has been reported that *lef-10* of BmNPV is required for its viral replication [23,24]. To investigate the role of LEF-10 in AcMNPV infection, a *lef-10* knockout AcMNPV bacmid (BacmidΔ*lef10*) was generated using the ET-recombination system in *E*. *coli* (Fig 1A). Co-transfection of *Sf*9 cells with BacmidΔ*lef10* and pTriEx/GFP failed to produce infectious baculovirus (Fig 2A left), in agreement with the previous reports that *lef-10* is essential for baculovirus replication [23].

To rescue viruses from BacmidΔ*lef10*, we re-introduced *lef10-GFP* driven by the *lef-10* intrinsic promoter, using pTriEx/lef10*p*:lef10-GFP, into the *polh* locus (Fig 1C). *Sf*9 cells were infected with the supernatant obtained from the transfected cell culture and observed at 72 hpi. Visible cytopathic effects (CPE) were observed under bright field (Fig 2A middle), demonstrating that the function of native *lef-10* was compensated by *lef10-GFP*.

As the expression level of LEF10-GFP under the control of its intrinsic promoter was quite low and hardly detectable by fluorescence microscope (Fig 2A middle), this made it difficult to characterize the protein in further studies. To increase the expression level of LEF-10, we used pTriEx/lef10-GFP to introduce *lef10-GFP* driven by tandem chicken actin promoter (recognized by cellular RNA polymerase II) and *p10* promoter into the BacmidΔ*lef10* (Fig 1D). Using this recombinant virus, the expression of LEF10-GFP could be initiated by the chicken actin promoter after virus infection, and the protein could also be over-produced under the control of the strong *p10* promoter in late infection. As shown in Fig 2A (the right images), infectious viruses were successfully rescued, and the over-expressed LEF10-GFP was also easily observed using the fluorescence microscope.

To investigate the subcellular localization of LEF-10, His-tagged LEF-10 under the control of the *lef-10* intrinsic promoter was introduced into BacmidΔ*lef10* to rescue the baculovirus, and the expression of His-tagged LEF-10 was detected by immunofluorescence assay using anti-His mAb and FITC conjugated secondary antibodies. Nuclear localization of the fluorescent protein were observed in most infected cells (Fig 2B), implying that LEF-10 may function in the nucleus.

### Identification of the essential region for LEF-10 function

To identify the LEF-10 functional region for the virus replication, a set of truncated *lef-10* gene fragments (*lef-10*_14–78_, *lef-10*_27–78_, *lef-10*_1–39_, *lef-10*_1–47_, *lef-10*_1–48_ and *lef-10*_1–52_, illustrated in Fig 1F) were inserted into pTriEx/GFP between *Nco*I and *Bam*HI sites, and then introduced into the *polh* locus to repair BacmidΔ*lef10* by homologous recombination. *Sf*9 cells were infected with supernatants obtained from the transfected cells and observed by fluorescence microscope at 72 hpi. Visible CPE and green fluorescence were observed in the cells infected with the recombinant viruses expressing *lef-10*_1–48_ and *lef-10*_1–52_, but not in the cells infected with the recombinant viruses containing *lef-10*_14–78_, *lef-10*_27–78_, *lef-10*_1–39_ and *lef-10*_1–47_ (Fig 2C). For those samples gave negative results, the supernatants obtained from the transfected cells were used to infect *Sf*9 cells for 3 passages, and still no CPE were observed. These results demonstrated that amino acids 1 to 48 were sufficient to recover the function of LEF-10 and the C-terminus (aa 49–78) of LEF-10 was not essential for the production of infectious viruses.

Growth kinetic analysis of LEF-10 repaired viruses confirmed that knockout of *lef-10* was lethal for AcMNPV, and the function of LEF-10 could be repaired by LEF10-GFP. Although over-expressing of LEF10-GFP and LEF10_1-48_-GFP could rescue the recombinant viruses, the virus titers decreased dramatically (Fig 2D). The replication level of the virus expressing truncated LEF10_1-48_-GFP reduced two orders of magnitude compared to LEF10-GFP, indicating that the C-terminus (aa 49–78) of LEF-10 was not essential for the producing infectious viruses but was beneficial for the virus production at high levels.

### Aggregation of LEF-10 in baculovirus infected cells

**Fig 3:**
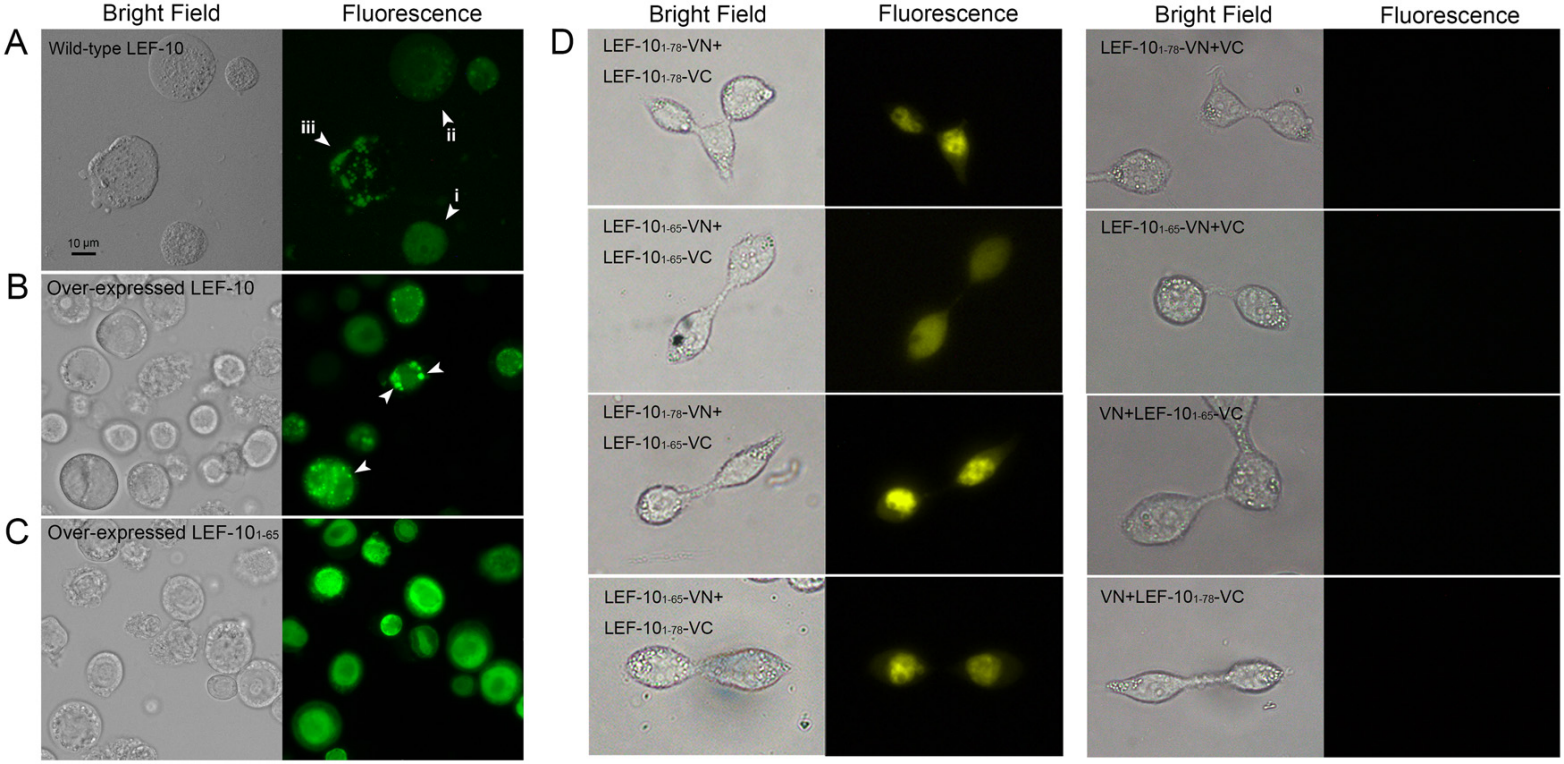
(A) Expression of LEF10-GFP fusion protein driven by *lef-10* promoter in *Sf*9 cells. Arrowheads indicated different status of LEF-10 in infected cells. i: evenly distributed, ii: aggregating and iii: aggregated LEF10-GFP. The cells were observed by florescence microscope (Leica DM5000B upright microscope) at 24 hpi. (B) Over-expression of LEF10-GFP or (C) LEF10_1-65_-GFP fusion proteins in *Sf*9 cells. The over-expressed full-length LEF10-GFP aggregated in more cells than the protein expressed using *lef-10* intrinsic promoter. The green fluorescence of LEF10_1-65_-GFP was evenly distributed in comparison with full-length LEF10-GFP (Obviously punctate dots indicated by arrowhead in (B) were not found in (C)). The cells were observed by florescence microscope at 48 hpi. (D) Detection of interactions between LEF-10 proteins in HEK 293T cells by BiFC assay. The cells were observed by florescence microscope at 24 hours post transfection of the indicated constructs.

To investigate the aggregation of LEF-10 in insect cells, the natural host of baculoviruses, the recombinant baculovirus expressing LEF10-GFP driven by its intrinsic *lef-10* promoter (Fig 1C) was used to infect *Sf*9 cells. LEF10-GFP was observed to aggregate in punctate dots in some infected cells (less than 1%), although the protein was uniformly distributed in the majority of the infected cells at 24 hpi (Fig 3A). Immunofluorescence assay using anti-His mAb showed that LEF10-His, the protein fused with a much smaller tag than GFP, also formed punctate dots similar to the LEF10-GFP fusion protein (Fig 2B). The results here suggested that the aggregation of LEF-10 could be a natural process in baculovirus infected cells. As no strong DAPI signals were found colocalized with the punctate dots (Fig 2B), it indicated that LEF-10 complexes were not stainable with DAPI and the aggregation of LEF-10 was not associated with apoptotic bodies which could easily be stained by DAPI.

We also observed that over-expressed LEF-10 driven by the *p10* promoter aggregated more frequently (about a quarter to a half of the cells) than the LEF-10 expressed under the control of its intrinsic promoter (Fig 3B). In comparison, the truncated protein LEF-10_1–65_ failed to form punctate dots in *Sf*9 cells even when it was over-produced under the control of *p10* promoter (Fig 3C). Deletion of the C-terminal region disrupted the aggregation of LEF-10 in infected cells (LEF-10_1–65_ in Fig 3C), but did not interrupt the protein’s function in virus replication (the replication level of the virus expressing LEF-10_1–65_ detected by virus titration was comparable to the virus expressing full-length LEF-10, data not shown), suggesting that aggregation of LEF-10 was not essential for the viral replication.

To further investigate the aggregation tendency between full-length LEF-10 and LEF-10_1–65_, bimolecular fluorescence complementation (BiFC) assays were employed in HEK 293T cells (Fig 3D). Out of our expectation, both full-length LEF-10 and truncated LEF-10_1–65_ interacted with themselves, respectively. Although LEF-10_1–65_ could interact with itself, the yellow fluorescence signal was more uniform and weaker compared with the self-interacted LEF-10 and LEF-10 interacted LEF-10_1–65_, indicating that the full-length LEF-10 was more prone to aggregation.

### Aggregation of LEF-10 down-regulated its function

**Fig 4:**
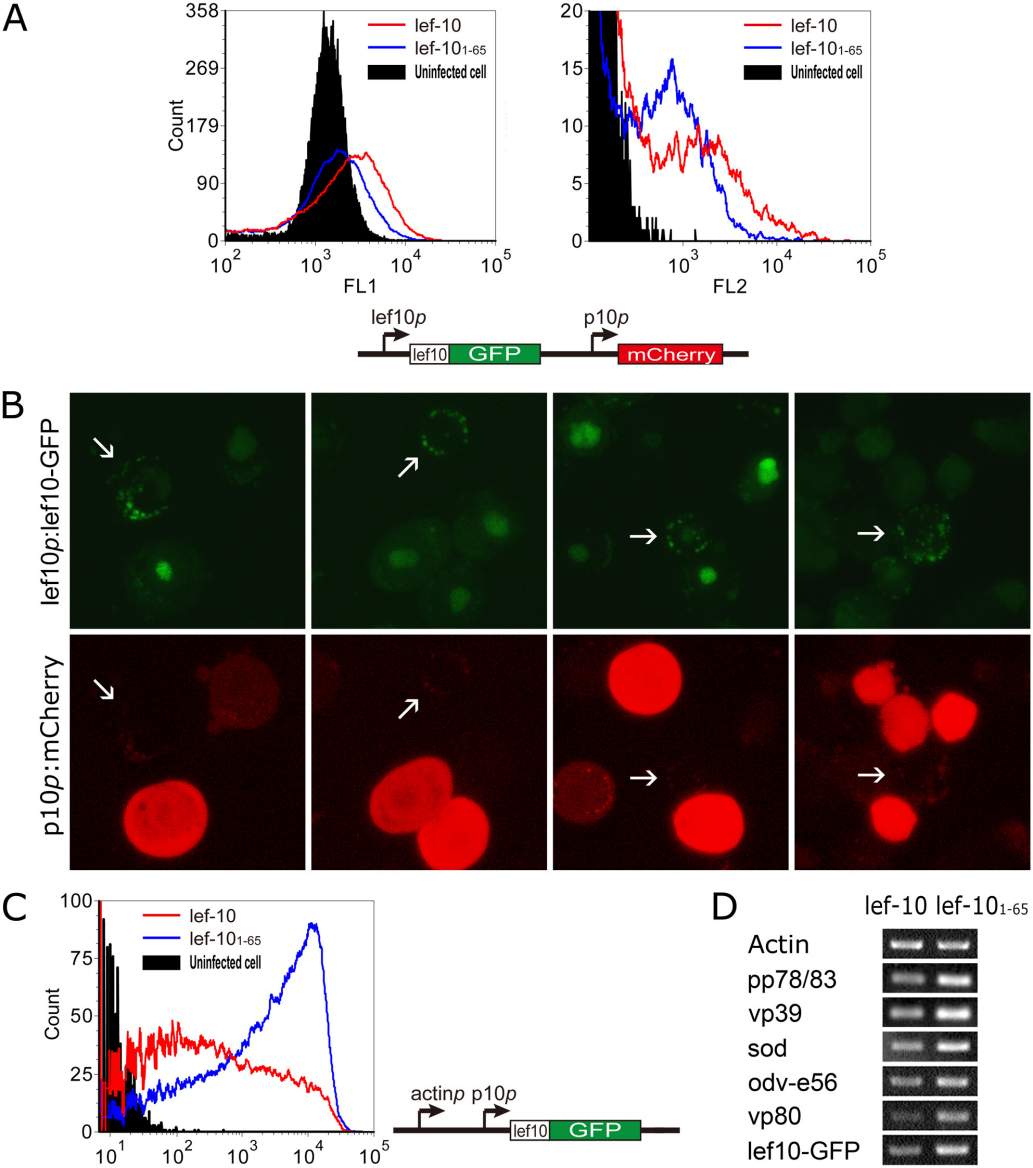
(A) Flow cytometry analysis of the effect of LEF-10 C-terminal truncation on the activity of *p10* promoter. *Sf*9 cells were respectively infected with vAcΔlef10/lef10*p*:lef10-GFP/p10*p*:mCherry or vAcΔlef10/lef10*p*:lef10_1-65_-GFP/p10*p*:mCherry and analyzed at 48 hpi. The expression of LEF10-GFP driven by *lef-10* promoter was detected in the FL1 channel, and mCherry expression controlled by the *p10* promoter was detected in the FL2 channel. The schematic diagram of expression cassette is at the bottom. (B) Fluorecence microscopic analysis of the correlation between LEF-10 aggregation and the expression of mCherry under the control of *p10* promoter. When the LEF-10 aggregation was observed, the red fluorescence was almost undetectable. The cells were analyzed at 48 hpi. (C) Flow cytometry analysis of the effect of LEF-10 C-terminal truncation on LEF-10 over-expression. *Sf*9 cells were respectively infected with vAcΔlef10/actin*p-*p10*p*:lef10-GFP and vAcΔlef10/actin*p-*p10*p*:lef10_1-65_-GFP, and the expression of the GFP fusion proteins, controlled by tandem chicken actin promoter and *p10* promoter, was detected in the FL1 channel. The schematic diagram of expression cassette is at the right site. (D) Analysis of the expression of five late genes, *pp78/83*, *vp39*, *sod*, *odv-e56* and *vp80* by semi-quantitative RT-PCR, in parrallel with the very late *p10* promoter driven lef10-GFP. Insect cell *β-actin* was used as internal reference. The left sample was from vAcΔlef10/actin*p-*p10*p*:lef10-GFP infected cells, and the right sample was derived from vAcΔlef10/actin*p-*p10*p*:lef10_1-65_-GFP infected cells.

To explore if the aggregation of LEF-10 was related to its function, two recombinant baculoviruses, vAcΔlef10/lef10*p*:lef10-GFP/p10*p*:mCherry and vAcΔlef10/lef10*p*:lef10_1-65_-GFP/p10*p*:mCherry, were constructed (Fig 1E). In these recombinant viruses, expression of LEF-10 or its truncated form was controlled by its intrinsic promoter, and mCherry driven by the *p10* promoter was used as a reporter to reflect the very late gene expression. Cells were infected in parallel with the two viruses at an MOI of 3 and collected at 48 hpi. Flow cytometry analyses showed that the fluorescence signal of both GFP and mCherry in vAcΔlef10/lef10*p*:lef10_1-65_-GFP/p10*p*:mCherry infected cells were slightly lower than the cell with vAcΔlef10/lef10*p*:lef10-GFP/p10*p*:mCherry (Fig 4A right), indicating that LEF-10_1–65_ could be expressed at a lower level under the control of its intrinsic promoter or less stable than the wild-type LEF-10 and it only partially recovered the function of full-length LEF-10 (Fig 4A left).

Observation of vAcΔlef10/lef10*p*:lef10-GFP/p10*p*:mCherry infected *Sf*9 cells by fluorescence microscopy at 48 hpi revealed that cells which formed punctate dots could hardly produce detectable red fluorescence signals (Fig 4B). In contrast, mCherry was abundantly expressed in the cells with LEF10-GFP evenly distributed (Fig 4B). This observation indicated that aggregation of LEF-10 down-regulated the expression of viral very late gene *p10*. Also, we observed that LEF-10 formed punctate dots predominantly located in the cytoplasm while non-accumulated LEF-10 mainly located in the nucleus (Fig 4B), indicating that LEF-10 functioned in the nucleus and aggregation of the protein in the cytoplasm probably depleted soluble LEF-10 and impeded the transportation of the protein to the nucleus.

Observed by microscopy, over-expressed LEF-10 aggregated more frequently than the LEF-10 expressed under the control of its intrinsic promoter in *Sf*9 cells but LEF-10_1–65_ did not aggregate to form punctuate dots even when over-expressed (Fig 3B and 3C). To further investigate the relationship between LEF-10 aggregation and its activity, cells infected with vAcΔlef10/actin*p-*p10*p*:lef10-GFP and vAcΔlef10/actin*p-*p10*p*:lef10_1-65_-GFP (the same viruses as in Fig 3B and 3C), respectively over-expressing wild-type LEF-10 and truncated LEF-10_1–65_, were further analyzed by flow cytometry. The expression level of LEF10-GFP in vAcΔlef10/actin*p-*p10*p*:lef10-GFP infected cells was obviously lower than that in vAcΔlef10/actin*p-*p10*p*:lef10_1-65_-GFP infected cells (Fig 4C), and this result was confirmed by semi-quantitative RT-PCR (Fig 4D bottom) at the transcriptional level, but was contrary to the protein expression levels under the control of *lef-10* promoter (Fig 4A). As LEF-10_1–65_ was detected at a lower level than LEF10-GFP when the protein expression was controlled by the weak *lef-10* promoter, these data suggested that the aggregation of over-expressed LEF10-GFP, but not LEF-10_1–65_, contributed to the down-regulation of LEF-10 function involved in the very late gene expression. Semi-quantitative RT-PCR analyses of other five late genes, *pp78/83* [29], *vp39* [30], *sod* [31], *odv-e56* [32] and *vp80* [33], indicated that LEF-10 aggregation affected the transcription of these late genes as well as the very late *p10* promoter controlled gene (Fig 4D).

## Discussion

LEF-10, the smallest late expression factor of baculovirus, contains only 78 amino acids and was originally identified in transient-expression assays as an essential factor for AcMNPV late promoter transcription [34]. In a previous report, we found that AcMNPV LEF-10 could form very stable SDS-resistant complexes [25]. A recent study using gene knockout bacmid has shown that BmNPV LEF-10 is indispensable for the viral DNA replication and viral gene transcription [23]. However, so far, LEF-10 has not yet been extensively studied, and the structure and the specific role of the protein in virus infection still remains unknown.

Here, using the similar strategy in BmNPV [23], we knocked out *lef-10* in AcMNPV bacmid and generated BacmidΔ*lef-10*. The BacmidΔ*lef-10* lost the ability to produce infectious virus and re-introduction of *lef-10* at the *polh* locus repaired its function in virus infection, demonstrating that *lef-10* is also essential for AcMNPV replication. Although no typical nuclear localization signal (NLS) was found in LEF-10 sequences, nuclear localization of the protein was observed in most virus infected *Sf*9 cells. Its nuclear localization is consistent with its classification as a regulation factor involved in late gene expression, and also suggests the protein’s probable role in late gene transcription.

**Fig 5:**
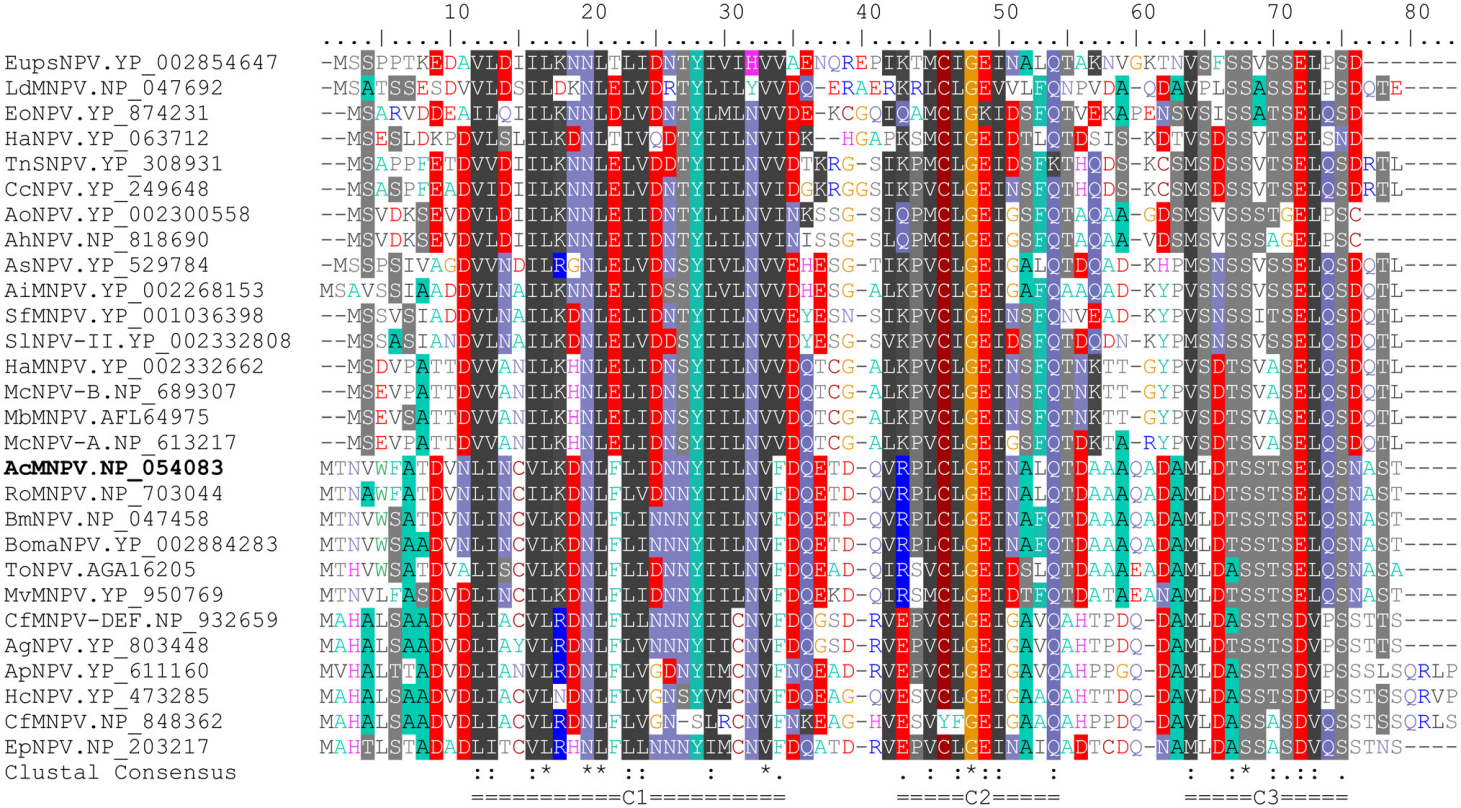
Amino acid sequence alignment of the LEF-10 of 28 nucleopolyhedroviruses. The alignment is produced using the CLUSTALW program [43]. Virus names and database access numbers are displayed on the left. Three constant regions are indicated at the bottom.

Using truncated LEF-10 to repair BacmidΔ*lef-10*, we defined the shortest functional LEF-10 moiety in the amino acids 1–48. Sequence alignment of AcMNPV LEF-10 with 28 NPVs identified three relatively conserved regions (C1, C2 and C3) in the protein (Fig 5). Our data showed that removal of part of the C1 region containing two conserved amino acids (L12 and I13) in LEF-10_14–78_ caused the disruption of its function in virus infection. Similarly, when the C2 region was partially removed (LEF-10_1–47_), repaired virus could not be generated. In contrast, the C3 region can be completely removed without affecting the virus survival. All these data indicate that the conserved C1 and C2 regions are important for the function of LEF-10 while the C3 region of LEF-10 is dispensable for AcMNPV replication. Further studies are needed to investigate whether it is the same case for other baculoviruses.

A number of proteins, including amyloid β-protein (Aβ) [35,36], sup35 [37], TAR DNA-binding protein (TDP-43) [38], α-synuclein [39] and mitochondrial protein MAVS [40], have been reported to be able to form SDS-resistant aggregates. However, their aggregated forms can be disrupted and seem not so stable as LEF-10. Although different expression and purification strategies have been tried, we are still unable to obtain purified LEF-10 monomers. Immunization of rabbit with the purified LEF-10 complex also failed to induce significant immune responses against LEF-10. These intriguing properties bring difficulties to further characterize this protein.

**Fig 6:**
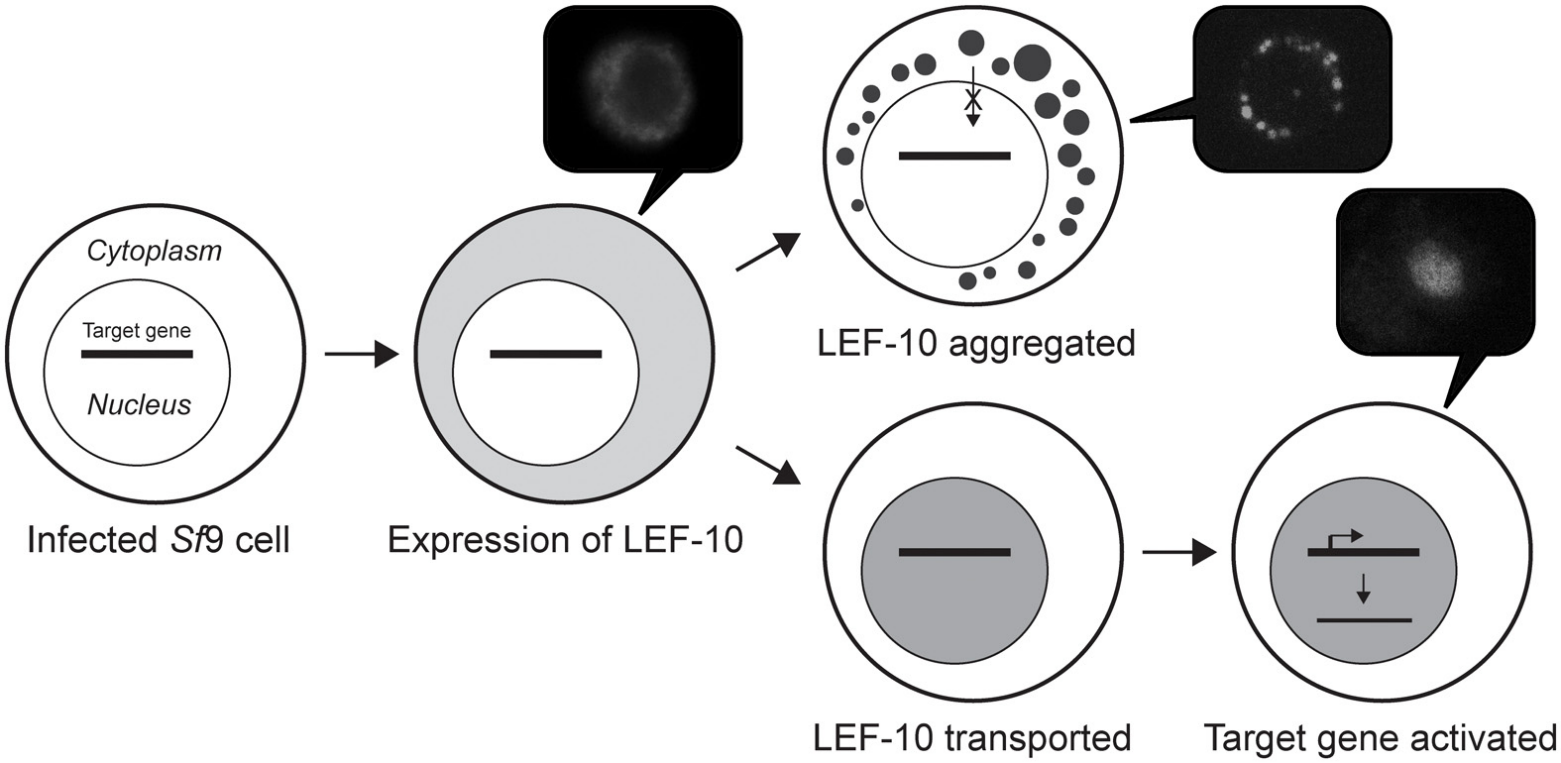
Model for LEF-10 aggregation and function in baculovirus infected cells. At early stage of infection, LEF-10 was translated in cytoplasm. In most cells, LEF-10 is transported into the nucleus where it can activate the expression of target genes. Meanwhile, over-production or some intrinsic unknown factor can trigger LEF-10 aggregation in some infected cells, and the aggregation may deplete the majority of soluble LEF-10 and impeded the protein’s translocation into the nucleus. Lack of LEF-10 in the nucleus results in the block of the activation of downstream target genes.

We believe that the aggregation nature of LEF-10 plays some role in virus life cycle. Using over-expressed LEF-10 and LEF-10_1–65_, we found that LEF-10 aggregation down-regulated the very late gene expression controlled by *p10* promoter. When LEF-10 was sequestered in punctate dots which surrounded the nucleus, the expression level of mCherry driven by the *p10* promoter was almost undetectable (Fig 4B). It seems that LEF-10 aggregation impeded its transportation from the cytoplasm to the nucleus, and therefore blocked its function in the activation of late gene expression. Based on these observations, a model for the relationship between LEF-10 aggregation and its function was established and illustrated in Fig 6.

Protein aggregation is a biological phenomenon mainly caused by protein folding or misfolding. Protein aggregation is often harmful and has been implicated in a wide variety of diseases. In *Saccharomyces cerevisiae*, aggregation of Sup35p will lead to the loss of its function as the release factor [41]. In a *Drosophila* model, cytoplasmic aggregation of huntingtin protein blocks axonal transport [42]. In the present study, we showed that LEF-10 aggregation down-regulated the expression of some late/very late genes. Sequence analysis showed that 47% amino acid residues in the LEF-10 sequence are hydrophobic, and most of the conserved residues, especially those in the C1 region, are hydrophobic amino acids (Fig 5). When the protein misfolding occurs, these hydrophobic amino acids may be largely displayed on the protein surface, causing the aggregation of LEF-10. It is an interesting question for further investigation whether these conserved hydrophobic amino acids are positively selected during virus/host co-evolution.
